# Drivers of Microbiome Biodiversity: A Review of General Rules, Feces, and Ignorance

**DOI:** 10.1128/mBio.01294-18

**Published:** 2018-07-31

**Authors:** Aspen T. Reese, Robert R. Dunn

**Affiliations:** a Society of Fellows, Harvard University, Cambridge, Massachusetts; b Department of Applied Ecology, North Carolina State University, Raleigh, North Carolina; c Natural History Museum of Denmark, University of Copenhagen, Copenhagen, Denmark; d German Centre for Integrative Biodiversity Research (iDiv), Leipzig, Germany; University of Hawaii at Manoa

**Keywords:** diversity, gut, microbiome

## Abstract

The alpha diversity of ecologic communities is affected by many biotic and abiotic drivers and, in turn, affects ecosystem functioning. Yet, patterns of alpha diversity in host-associated microbial communities (microbiomes) are poorly studied and the appropriateness of general theory is untested. Expanding diversity theory to include microbiomes is essential as diversity is a frequently cited metric of their status. Here, we review and newly analyze reports of alpha diversity for animal gut microbiomes. We demonstrate that both diet and body size affect diversity in the gut but that gut physiology (fermenter versus simple) is the most important driver. We also assess the advantages of various diversity metrics. The importance of diversity in microbiomes is often assumed but has not been tested outright. Therefore, we close by discussing how to integrate microbiomes into the field of biodiversity-ecosystem functioning to more clearly understand when and why a host supports diverse microbial communities.

## Abstract (precis)

The alpha diversity of ecologic communities is affected by many biotic and abiotic drivers and, in turn, affects ecosystem functioning. Yet, patterns of alpha diversity in host-associated microbial communities (microbiomes) are poorly studied and the appropriateness of general theory is untested.

## PERSPECTIVE

Diversity, one of the most widely studied ecologic properties, is fundamental to the field of ecology itself. It is widely reported and often promoted as an indicator of ecosystem state due to its relationships with productivity, functioning, and stability (1–4). However, diversity is not an inherent feature of communities, nor is it a universal good. Specifically, more diversity is not always better (5–8). Therefore, diversity cannot be assessed in a universal framework but instead must be contextualized or relativized within the ecosystem of interest. The difficulties of interpreting diversity, both its drivers and consequences, are particularly acute when considering microbial systems.

To date, the majority of research into patterns of diversity and biodiversity-ecosystem function has been carried out in plant communities or with vertebrates at larger spatial grains. In these macroscopic communities, diversity has long been documented to vary predictably with geographic area, latitude, altitude, time, and disturbance, among other factors (9). Major research efforts to build diversity-function relationships from this literature began in the early 1990s and have continued unabated since then (10, 11). While the diversity of some microbial communities has also been shown to vary predictably along gradients or in response to disturbance (12–17), only a small proportion of the diversity-function literature addresses microbial eukaryotic or bacterial communities (but see references 18, 19, and 20) even though most species are microbes (21). An interesting exception is work on the importance of evolution to the dynamics of biodiversity. Because experimental evolution is more tractable for analyses of microbes, they are more often used for experiments studying diversity and evolution conjointly (see, for example, references 22, 23, and 24), demonstrating that such experiments are possible and even uniquely valuable for testing ecologic theories. Relatively little work has been done to bridge the divide between large and small (see, for example, reference 21). This dearth may in part reflect differences in biology and even physics between macroscopic and microbial systems, but we suspect that it just as often reflects differences in the scientific cultures of those who study “macrobes” and those who study microbes.

The lack of relevant microbial theory is particularly apparent when considering host-associated microbial systems, i.e., microbiomes. Diversity metrics are frequently reported in amplicon sequencing-based assessments of communities, and variations in diversity are calculated between hosts or between case and control states. Without a theory of diversity applicable to microbiomes, however, it is hard, if not impossible, to interpret these results. Even in parasites, for which there is a much longer tradition of research, there has been only limited work on patterns of diversity inside hosts (see, for example, references 25 and 26) rather than globally irrespective of individual animals (see, for example, reference 27). The patterns of microbiome diversity, pathogenic or commensal, among individuals, within host species, or between host species have not been systematically described. Without such knowledge, the obstacles preventing our understanding when changes in diversity are important remain insurmountable.

Based on what has been found in plant, animal, and free-living microbial communities, we would expect diversity in microbiomes to be impacted by a number of factors. Factors which increase niche availability, such as diverse resources or increased area, or which limit growth, such as frequent disturbance or chronically extreme conditions, are expected to drive diversity in opposite directions. The specific forms of these factors likely differ depending on the body site considered—for example, disturbances to the skin could include bathing or variation in environmental temperature, whereas the gut would be more susceptible to ingested pharmaceuticals or changes in diet. Similarly, the levels of importance of microbial community diversity to the host are also likely to differ between body sites. Here, we focus on differences among species in the diversity of the gut microbiota, which is arguably the most highly studied host-associated microbial community type.

Drawing on previous reports, we suggest that gut microbial community diversity is positively associated with diet diversity and the proportion of calories derived from plant material and that increases in both should increase the number of available nutritional niches. We also suggest that gut microbial community diversity tracks body size, which increases available gut space (akin to increases in area in other ecosystems) and transit time. Physiological features such as stomach pH, which influences the ability of microbes to colonize the gut, and gut morphology, which alters available habitat and residence time, may also contribute. To test these hypotheses, we reviewed the literature and conducted new analyses of data drawn from previously published studies. We then discuss our limited current understanding of diversity-ecosystem functioning in the gut and propose future work to address this limitation.

## VARIATION BETWEEN ANIMALS

Few studies have explicitly tested the drivers of variation in bacterial (or, more generally, microbial) community diversity in guts among animal species. In general, these studies have tended to suggest an effect of diet on gut microbial community diversity, with herbivory associated with higher diversity. Among New World bats, the phylogenetic diversity of gut bacteria was found to be associated with dietary strategy; phylogenetic diversity was greatest in fruit-feeding bats and lowest in blood-feeding bats (28). More recently, Godon et al. (29) found that diversity was generally higher in herbivores but that the effect of diet was mediated by body size, with an overall positive relationship between body mass and gut bacterial community diversity (measured using the Simpson index) across a cohort of distantly related animals in captivity (including birds, mammals, and reptiles). Captivity itself has also been found to affect gut diversity in some mammals (30), but the relationship did not have a consistent signal (30, 31).

Heuristic comparisons among taxa and contexts reveal patterns that support our hypothesis that diet diversity and gut morphology are important drivers of gut microbial community diversity. Small insects with simple diets often have low-diversity communities. Caterpillars, which have short gastrointestinal transit times and short guts, harbor very low microbial community diversity, driven by species sourced from the food plant material itself (32). Fruit flies and honeybees similarly carry only a few species of bacteria consistently in their guts (33, 34). In the case of *Drosophila*, individuals can lose their microbiota in the absence of replenishment from bacterially colonized food (35) although there is also evidence that some bacteria can persist and grow when added to an otherwise axenic gut (36). At the other end of the spectrum, humans living more traditional lifestyles, which are associated with higher-fiber diets and longer transit time, have been found to have higher-diversity gut microbiota than do those living Westernized lifestyles (37, 38). Interestingly, there is not a significant difference in most diversity metrics between Inuit individuals who consume traditional but low-fiber diets and Canadians living in a city (39). This result suggests that it is not traditional lifestyles *per se* that necessarily lead to more diverse gut microbes but instead certain kinds of diets.

To test specific hypotheses of a general model of diversity in host-associated communities, we conducted a review of relevant literature on the gut microbiota. Gut microbiota are important for nutrient and waste processing, signaling, and immune function (40–44). Gut microbes are also, in most cases, among the most diverse host-associated communities (45). The field of gut microbiome research has grown exponentially over recent decades, including, but especially since, the decade in which the NIH Human Microbiome Project began. Therefore, we expected it to have the largest data sets to draw from. We conducted a literature search in Google Scholar for “gut AND diversity AND microb*” and also read papers cited in the work found through the search. We focused on 16S rRNA gene amplicon data and on healthy individuals found both in the wild and in captivity. We then extracted data from the published text and figures of 36 studies (see Table 1; see also Data Set S1 in the supplemental material). Data found only in figures were estimated using GraphClick, which is graph digitizer software. We used the authors' own definition of a taxonomic group (typically, species or operational taxonomic unit [OTU]), which in most studies, as in most of the literature, was 97% similarity of the 16S rRNA gene, although other cutoff values were also used (see, e.g., references 46 and 47) or not specified (see, e.g., reference 48). In cases where host species were represented in multiple publications from the same population, we included only data from the study with greater sequencing depth (see, for example, references 49 and 50). If averages of sequencing depth data or rarefaction depth data were not reported, we used the lower end of the range reported. In some studies, no information was reported on sequencing depth. In two studies (28 and 30), data were available only at the family level. We then collected metadata on animal diets and body size for a subset of animals from a comprehensive database for mammals and birds (51). Gut structure (ruminant, hindgut fermenter, simple gut) data were available in many publications that we included but were also corroborated for additional species through targeted literature searches.

10.1128/mBio.01294-18.1 DATA SET S1  Data extracted from studies and included in this study. "Other_notes" specifies the diversity metric noted under the “Other” column. "Diet notes" includes any information from the publication about the diet of the organism. Additional diet information and body mass data are from reference 51. pH data are from reference 55. Gut length data are from references 52, 53, and 54. Download DATA SET S1, CSV file, 0.03 MB. Copyright © 2018 Reese and Dunn. 2018 Reese and Dunn This content is distributed under the terms of the Creative Commons Attribution 4.0 International license.

**TABLE 1:** Summary of studies and taxa included in this analysis^a^

Citation	Taxon(s)	Diversity metrics reported	No. of taxa with indicated data reported		
			Gut structure	Gut length	pH
Aivelo et al. 2016 (105)	Mouse lemur	R, IS			
Bennett et al. 2013 (106)	Emu	Sh, C, Si			
Bletz et al. 2016 (107)	Fire salamander	Ch, PD			
Borbón-García et al. 2017 (108)	Andean bear	Sh, C, Si	2		
Carey et al. 2013 (82)	Thirteen-lined ground squirrel	R, PD			
Carrillo-Araujo et al. 2015 (109)	6 bat species	R, Sh, PD	1	1	
Chandler et al. 2011 (110)	13 wild fruit fly species	R, Sh, C, PD			
Clemente et al. 2015 (37)	Human (4 populations)	PD			
Dewar et al. 2013 (111)	4 penguin species	Sh			2
Ding et al. 2017 (112)	Chinese mitten crab	R, Sh, Si			
Dubois et al. 2017 (39)	Human^b^ (2 populations)	R, Sh, C, Si	1	1	1
Givens et al. 2015 (113)	15 fish species	R, Sh, C, PD			
Gruninger et al. 2016 (114)	Beaver	R, C, PD	1	1	2
Hale et al. 2018 (31)	5 primate species	R, Sh, C, PD	3	1	1
Handl et al. 2011 (48)	Dog, cat	R, Sh, SR	1	2	2
Keenan et al. 2013 (46)	Alligator	Sh, C			
Kohl et al. 2017 (115)	3 iguana species	R, Sh, PD			
Ley et al. 2008 (49)	24 mammal species	R	21		3
Li et al. 2016 (116)	Plateau pika, Daurian pika	R, PD	2		
Lin et al. 2013 (117)	Human (2 populations)	R, Sh, C, PD			
Matsui et al. 2010 (47)	Ostrich	Sh			
McKenney et al. 2015 (118)	3 lemur species	Sh			
McKenney et al. 2017 (68)	Giant panda, Red panda, Bamboo lemur	Sh, C	3		
McKenzie et al. 2017 (30)	5 mammal species, 7 mammal families	Sh	9	1	
Metcalf et al. 2017 (65)	Przewalski's horse, domestic horse	Sh	2	1	2
Muegge et al. 2011 (50)	31 mammal species	R, Sh	30	10	8
Phillips et al. 2012 (28)	7 bat families	PD			
Raymann et al. 2017 (85)	Honeybee	Sh			
Roggenbuck et al. 2014 (119)	Turkey vulture, Black vulture	Sh			1
Schnorr et al. 2014 (38)	Human (2 populations)	R, Sh, C, PD			
Smits et al. 2017 (80)	Human (2 populations)	R, PD			
Stevenson et al. 2014 (120)	Arctic ground squirrel	R, C, PD			
Sullam et al. 2015 (121)	Trinidadian guppy	R			
Wu et al. 2017 (122)	Panda	R, Sh	3		
Xie et al. 2016 (123)	Red-crowned crane	R, Sh, C, PD			
Yildirim et al. 2010 (124)	Red-tailed guenon, Red colobus	R, Sh, C, E	2		

a Diversity metric abbreviations: R, richness; IS, inverse Simpson; C, Chao1; Sh, Shannon; Si, Simpson; PD, phylogenetic diversity; SE, Shannon’s evenness; SR, Simpson’s reciprocal index; E, evenness.

b Data on a Western, modern human population were used to stand in for humans for most analyses to prevent pseudoreplication.

We first tested for individual effects of taxonomic class (e.g., mammal, bird, or insect; *n =* 259), diet class (carnivore, omnivore, or herbivore; *n =* 259), and gut physiology (foregut ruminant, hindgut fermenter, or simple gut; *n =* 176) on diversity using a combination of Kruskal-Wallis and Mann-Whitney *U* tests. Multiple-hypothesis correction (Bonferroni correction) was applied to any comparison with more than three levels. To explore the interaction between diet and physiology, we analyzed a subset of mammalian fecal samples for which data on diet and physiology were available (*n =* 63 to 67) using linear mixed-effects models that included diet or physiology or diet and physiology as a fixed effect(s). All models included sequencing depth as a fixed effect and publication identity (i.e., which publication the data came from) as a random effect. Likelihood tests were used for comparisons of the models to one another and to a null model that included only sequencing depth. Similarly, we tested for an effect of body mass on diversity in mammalian fecal samples (*n =* 52 to 58) by comparing a linear mixed-effects model that included body mass and physiology to one that included only physiology. Again, sequencing depth and publication identity were included in both models. These analyses were carried out in the “lmer” package in R. Finally, we were able to extract more-detailed physiological data on gut length (52–54) and stomach pH (55) for a subset of animals. Gut length data were included in cases where data for members of the same genus were present in both data sets. Gut length (*n =* 15) and pH (*n =* 14 to 16) were analyzed with partial Spearman correlations which corrected for variations in sequencing depth. We included publication as a random effect or sequencing depth as a covariate in our analyses in order to correct for differences in sequencing approaches between studies. Not all studies were included in all analyses, because not all data were available for each parameter and, notably, because not all studies reported sequencing depth.

### Shannon diversity and richness.

**FIG 1:**
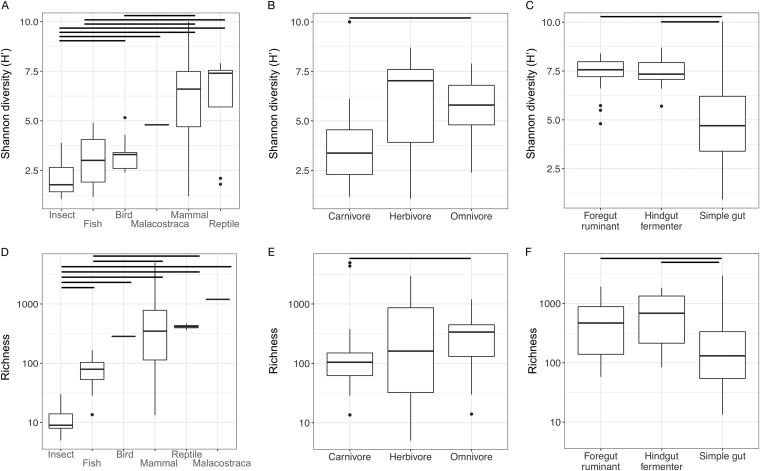
Individual drivers of gut microbiota diversity in animals. Gut Shannon diversity values differ between some taxonomic groups (A), diet types (B), and gut physiologies (C), although there is no overall effect of these variables on diversity. Similarly, richness values differ between some taxonomic groups (D), diet types (E), and gut physiologies (F) without overall effects. Horizontal lines indicate pairs which differ significantly (*P* < 0.05 [Mann-Whitney *U* tests]). Box plots show means and quartiles.

**FIG 2:**
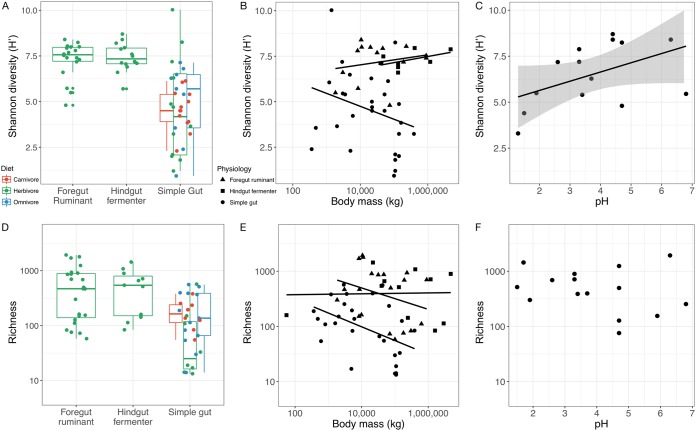
Drivers of gut microbiota diversity in mammalian fecal samples. (A to C) Gut Shannon diversity is associated with physiology and diet (A), body mass (B), and stomach pH (C). (D to F) Gut richness is associated with physiology and diet (D) and body mass (E) but not pH (F). Box plots show means and quartiles. The body mass plots (B and E) show nonsignificant linear fits to illustrate the different trends between physiological groups which result in a stronger overall effect of physiology than of body size. The linear relationship between stomach pH and Shannon diversity (C) is plotted, with standard errors in gray.

Shannon diversity, a metric that weights the numbers of species by their relative evenness data, was the most commonly reported diversity metric. We found that Shannon diversity data varied among some taxonomic classes, diet groups, and gut physiologies (*P* < 0.05 [Mann-Whitney *U* tests]) but that there were no overall effects of any of these variables (*P* > 0.05 [Kruskal Wallis tests]; Fig. 1A to C). In mammalian fecal samples, Shannon diversity was associated with host diet but more strongly with gut physiology. Linear mixed-effects models that included diet class or gut physiology outperformed a null model that included only sequencing depth (*P* < 0.001 [likelihood tests]), but the model that included both diet class and physiology did not outperform the model that included only physiology and sequencing depth (*P* = 0.80). Fermenters, both foregut and hindgut, had higher gut microbial richness values than did animals with simple guts; the effect of the inclusion of ruminants led to a pattern wherein herbivores (a subset of which are ruminants) seemingly had higher diversity overall than omnivores or carnivores (none of which are ruminants; Fig. 2A). The diet effects disappeared in comparisons performed just within animals with simple guts. Shannon diversity was similarly associated with variations in body mass. A model that included body mass outperformed the null model (*P* = 0.03 [likelihood test]), but this effect did not result in greater performance than that seen with a model that simply included physiology (*P* = 0.90 [likelihood test]). The explanation for this result is likely that the relationships between body mass and diversity had different directions depending on host physiology (Fig. 2B)—as ruminants increased in body mass, their Shannon diversity increased, whereas animals with simple guts showed the opposite trend wherein the guts of larger animals with simple guts tended to be lower in microbial community diversity. This may be in part a consequence of the fact that large animals with simple gut physiology tend to be omnivores and carnivores.

Richness, i.e., the total number of OTUs or species recorded, was the second most commonly reported diversity metric and showed patterns similar to those seen with Shannon diversity. The richness data differed between some taxa, dietary groups, and physiological groups but did not show overall effects of these variables (*P* > 0.05 [Kruskal Wallis tests]; Fig. 1D to F). In mammals, richness was impacted by diet within animals with simple guts, but across the board, herbivorous hindgut or foregut fermenters had the highest diversity (Fig. 2D). Linear mixed-effects models that included diet class or gut physiology outperformed the null model (*P* < 0.001 [likelihood tests]), but the interaction model did not outperform the physiology model (*P* = 0.18). A body mass model outperformed the null model (*P* < 0.001 [likelihood test]), but, as with Shannon diversity, inclusion of the body mass term did not improve model performance relative to one that included physiology alone (*P* = 0.20 [likelihood test]; Fig. 2E). To the extent, then, that body mass matters, it seems to matter with respect to effects on the relative abundance and evenness of species rather than their total count.

Gut length measurements, which were available for a subset of taxa, were not associated with diversity (richness or Shannon diversity), even correcting for study sequencing differences (*P* > 0.05 [partial Spearman correlations]). Similarly, the pH of the stomach, which can act like a filter, preventing, in proportion to its acidity, the colonization of the gut by microbes in the years after birth (56), was not associated with richness (*P* = 0.31 [partial Spearman correlation]; Fig. 2F) and was associated with Shannon diversity only marginally (*P* = 0.05 [partial Spearman correlation]) (Fig. 2C). As gut length data were available for only 19 genera and pH data for 23 species, such relationships may require greater power to compensate for study-to-study variability.

### Other metrics.

Fewer than a third of animals had other estimates of diversity reported. These included phylogenetic diversity, Chao1, and, less commonly, Simpson’s diversity. Perhaps because of lower sample sizes, these metrics were not consistently associated with diet or physiology and were more often driven by large variations in sequencing depth.

**FIG 3:**
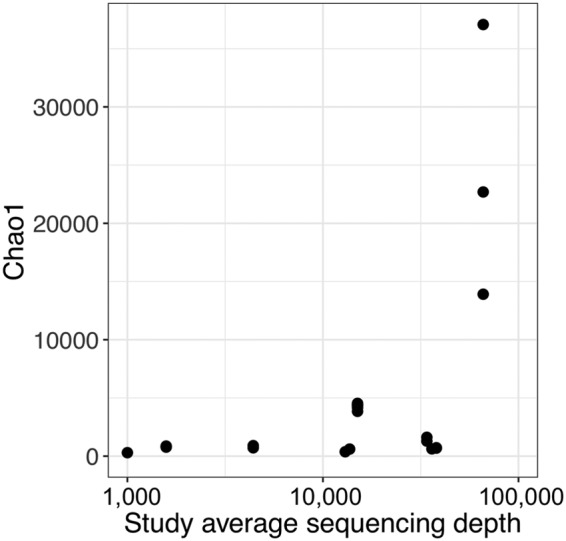
Variation in Chao1 diversity based on sequencing depth. One study with much greater depth (66,000) showed an increase in variation in diversity by an order of magnitude.

Because different research projects differ with respect to sequencing technology, target primers, and, perhaps most importantly, sequencing depth, diversity values can vary greatly for reasons decoupled from biology. Diversity is highly correlated with sequencing depth, i.e., the number of reads sequenced per sample, similarly to how diversity is correlated with sampling effort in studies of plants or animals (57). But the debate over whether or not to rarefy microbial sequencing data to a set diversity depth is ongoing (58). Chao1 is an estimator designed to specifically account for differences in sampling effort between studies (59) and should in theory be more robust in comparisons between microbial sequencing studies (60) (but see reference 61). In practice, the sensitivity to sequencing depth in our analysis was most apparent for Chao1 (Fig. 3), although this may also reflect differences in sequencing technologies. While it is possible that these metrics of diversity would yield greater insight if available from a broader swath of the literature, variations between samples with respect to such values are currently extremely difficult to interpret. Overall, our results, in conjunction with other reports on the difficulties in estimating microbial community diversity (62), indicate that the data from comparisons of Shannon diversity may be the most robust and informative, particularly given the frequency with which this metric is already reported in the field.

### How to improve these analyses.

In general, we find a coarse relationship between gut architecture and host diet with respect to gut microbial community diversity. There are, of course, many other factors which, we predict, could impact gut alpha diversity, but such data are harder to find consistently in the literature. Immune system complexity and functioning can together serve as a filter for specific taxa or microbes in general (63), but studies that consider the influence of immune complexity and function would require generally agreed-upon definitions and measurement protocols for immune function. A varied diet is expected to create more metabolic niches for the microbiota and thus to increase microbial community diversity (64). Estimates of diet diversity, specifically, of plant diversity, are difficult to calculate without observational or genomic measurements of diet, though. In our survey, sequencing-inferred diet data were available for only one study (65), which included just two host species, but such data could easily be collected in future studies through inclusion of plant DNA sequencing (66) in addition to the microbial sequencing. Finally, a short retention time in the gut is expected to lower diversity as it selects for only fast-growing taxa—similarly to how high disturbance rates are commonly associated with low diversity in plant communities (67). This trend has been shown for select host taxa (68) but has not been tested with a diverse group of hosts. Because body size is not necessarily a strong predictor of gastrointestinal transit time (69), actual estimates of transit time will be necessary to truly test this hypothesis more generally.

Overall, we found that the best predictor of gut microbial community alpha diversity variation among animals was the presence or absence of a simple gut. Ruminants and hindgut fermenters consistently supported higher levels of diversity than did species with simple guts. While herbivores are typically thought to have more diverse gut microbial communities, we found that herbivores with simple guts, such as some bears, tended to have diversity levels similar to those of predators. We hypothesize that this effect is due specifically to the gut architecture and, as an extension, the short transit times (68).

Such variations between herbivores on the basis of gut architecture lead to an important outstanding question in the study of microbiomes—does it matter for the host whether its microbial community is diverse? If diversity is important for function, herbivores with simple guts, such as giant pandas or spectacled bears (49), would be predicted to receive less benefit from their microbiomes and as such might experience evolutionary pressure for behavioral or physiological compensation. Alternatively, it may be that a diverse community is not necessary for digestion of plants and that the large, complex communities found in herbivorous foregut and hindgut fermenters primarily reflect the possibility of higher diversity in large, complex environments rather than the diversity’s adaptiveness for the host *per se*. Addressing this issue requires moving beyond measuring diversity to experimentally manipulating it, as discussed below.

## DOES VARIATION MATTER, THOUGH?

In order to address the issue of when microbial community diversity is important to hosts will require an understanding of diversity-function relationships in host-associated systems. Insofar as descriptions of ecosystem processes represent a human construct, they typically describe processes of value to humans. But it is also imaginable that diversity could be related to functions that are features of the microbial community which are independent of the host or even at odds with the fitness of the host. Both types of function (those of value to the host and those not) will need to be assayed to understand the role of diversity in and on hosts.

While it is clear that hosting microbial communities can be functionally beneficial or even necessary to an animal (70–73), the extent to which hosting a diverse microbial community is necessary for this phenomenon is still uncertain. To ensure specific, mutualistic interactions, hosts can just select for low-diversity, high-fidelity microbial partners such as those in the light organs of bobtail squid (74). Indeed, a host may wish to limit diversity because not all microbes are beneficial, whether because they are pathogens or because they are just cheating strains that avoid providing function to the host (75). Furthermore, increasing diversity has been shown to actually reduce stability in gut communities (76). As such, evolution has selected for many pathways for use by hosts to control their microbiota through the immune and digestive systems (63), which may be equally likely to be employed to select for high-diversity and low-diversity gut microbial communities. It may be that in many cases, hosts would prefer to maintain their microbial communities as humans keep farms—as low-diversity but high-function systems—but the long-term ecologic and evolutionary implications of this remain unclear.

Preliminary assessments of the role of diversity in the gut have focused on studying cases where diversity changes over time. As infants grow, the diversity of their fecal microbiota increases both within the first 6 months (77) and over the first few years (78, 79). How much of this trend is merely due to the increase in body size is not clear. Seasonal variation in diversity has been documented both in animals whose diets vary (80, 81) and in those which hibernate, with lower diversity during hibernation (82, 83). In all these cases, changes in diversity are assumed to be associated with changes in function, specifically, with shifts in metabolism, but functional measurements are rarely explicitly included. One alternative possibility is that variable environments experienced over time or space produce changes in the available species pool dispersing into hosts that manifest as variations in the community. In the absence of data on variations in functioning, it is impossible to interpret changes in microbial community diversity over time.

More generally, diversity is expected to decrease in disturbed systems. For example, treatment with antibiotics is often tied to a reduction in gut microbial community diversity (84–86). More generally, illness, which can trigger the use of antibiotics, is also assumed to reduce diversity or to even be the result of a loss of diversity. In fact, while the definition of dysbiosis continues to be debated (87), the inclusion of “loss of diversity” is one constant across all definitions. A recent meta-analysis of case-control studies of human disease calls this definition into question, however (88)—across 28 studies, there was no consistent reduction in diversity for sick patients relative to healthy individuals. In 13 cases, disease was accompanied by a loss in Shannon diversity, but the results were not always consistent across multiple studies of the same disease. A further 14 cases did not show a significant response in Shannon diversity, while one study of HIV found an increase in diversity in patients. Only in diarrheal illnesses (Clostridium difficile infection and enteric diarrheal disease) was there a consistent decrease in diversity, perhaps attributable to the decrease in transit time. In light of this result, it may be necessary to reconsider the definition of dysbiosis as well as more generally reconsider when and why we might expect variation in diversity to be tied to changes in function or vice versa.

### Future directions.

To the best of our knowledge, experiments to test for diversity-function relationships have not actually been carried out in host-associated systems. The impact of parasites on ecosystem functioning has been considered but only insofar as they impact overall diversity through numeric and behavioral effects on host species and not as constituent members of the community (89). Nevertheless, such experiments can conceivably be designed, and researchers can learn from and build on more-traditional ecologic work. As has been done for plant and animal communities, the impact of diversity can be tested either by removing community members to reduce diversity or by artificially assembling communities of increasing diversity (reference 90).

### (i) Experiments increasing diversity.

**FIG 4:**
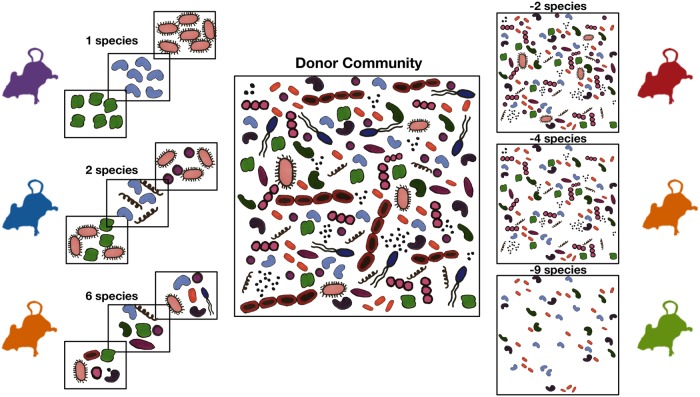
Experimental approaches to increasing or reducing diversity in host-associated communities. Increasing diversity from zero or reducing it from the whole-community level is expected to result in variations in functioning, which would inform our understanding of biodiversity-ecosystem functioning in the microbiota.

The majority of diversity-ecosystem functioning experiments, including foundational studies by Naeem et al. (1) and Tilman et al. (91), were carried out in artificially assembled communities. In such studies, individual plots are assigned a diversity level and then taxa are randomly selected from a regional species pool to colonize those plots (Fig. 4). Function data can be compared between treatment groups or on the basis of the presence of specific taxa.

Faith et al. (92) demonstrated an appropriate methodology using donor culture collections and germfree mice to assemble various microbial communities in the gut. Phenotypes were measured in mice colonized with randomly assembled communities. The work used communities with a consistent diversity level (17 strains), but the approach could be extended to build and test communities of various diversity levels. The automated processes involved allow studies of high-diversity communities that are not typically possible when the studies are carried out manually, overcoming a frequent criticism of synthetic community studies such as this.

For animals which do not have germfree lines, synthetic community functioning can be measured *in vitro* or in a host different from the donor species. It is common to colonize mice with communities from nonmouse donors, and such communities can mostly persist in the absence of dispersal of conventional community members (93, 94). The context dependency of taxon-specific host-microbe interactions would be missed in such setups, however.

### (ii) Experiments decreasing diversity.

Because many studies of disturbances to host-associated systems have been performed on the basis of an interest in loss of diversity, removal of taxa may more accurately reflect the target processes than the building of artificial communities with culture collections drawn from donor communities.

Instead, whole donor communities can be diluted to produce communities with reduced diversity (see, for example, the analysis of lake bacteria in reference 95). These reduced communities can then be used to colonize animal donors or studied *in vitro* alongside the whole donor community to assess how functioning is altered and whether the alteration is due to loss of specific taxa or to loss of diversity overall (Fig. 4). While dilution randomly removes individuals, it disproportionally affects rare taxa and therefore is not appropriate for studying their importance.

An alternate approach is that of performing removal experiments *in vivo*, which can test the influence of diversity in naturally assembled communities. This approach benefits from the fact it does not rely on germfree organisms, thereby ensuring that the host and microbiota experience a more conventional developmental process and that the experiments can be performed on a broader set of host animals.

Methods for targeted removal of microbial taxa represent an area of focused research and are becoming increasingly common. In addition to narrow-spectrum antibiotics, phage cocktails and gene editing technologies can be used to selectively alter community composition and diversity levels.

### Broader measurements.

In addition to devising new experimental designs, to properly assess biodiversity-ecosystem functioning in host-associated microbial communities, researchers will need to think more broadly about how they define functioning and how they define diversity. Multiple functions will need to be assayed in a range of organisms to validate the generalizability of the relationship, but the initial focus should be on testing the foundational metrics, including productivity, stability, and the invasibility of the community, as well as on measures of host fitness which do not have corollaries in the environmental literature (92–94).

Explorations of diversity-function relationships in host-associated systems will likely require measurements beyond microbiome profiling. Microbial biomass or concentration is rarely quantified but has been shown to provide a better interpretation of community changes than relative abundance data alone (96, 97). A conventional measurement of activity or productivity could also help researchers to better estimate the functional contingent of a diverse microbial community. While some functional measurements (e.g., survival of infection or severity of disease state) will be idiosyncratic, general protocols will be necessary to build a comprehensive theory of diversity-function relationships in host-associated systems that encompasses many hosts and many body sites.

Assessments of multiple taxonomic levels of diversity will also be necessary. Most studies have focused on species-level or OTU-level diversity (typically defined by 97% sequence similarity), but meaningful functional differences can exist at much higher or lower taxonomic levels (98). Horizontal gene transfer and lysogenic phage infection, for instance, can lead to single-gene differences or copy number variation between otherwise identical strains that may be functionally important for ecosystem functioning in the gut but remain uncaptured by typical amplicon sequencing-based analyses (33, 99–101).

## CONCLUSIONS

As we have come to study an increasingly broad swath of the tree of life, it has become clear that animals can get by and often have high fitness with low-diversity microbiota (75). Therefore, the framework for understanding diversity in host-associated microbial communities will not be as simple as “more diversity is better.” In addition to experimental tests in model organisms, further comparative work between organisms with consistent methodology will be necessary to determine the drivers of diversity in hosts. Additional comparisons between body sites could highlight other relevant factors such as the method of transmission or specific environmental conditions. The focus of ecologic studies has recently turned toward assessments of multifunctionality in diversity-function relationships (see, for example, references 102 and 103), and these relationships will ultimately need to be understood for host-associated microbial communities as well. Selection for maintaining a community that can provide multiple functions while preventing negative consequences of hosting microbial communities is an ongoing process driving evolution of both hosts and their commensal microbes (104).
